# High-resolution image inpainting using a probabilistic framework for diverse images with large arbitrary masks

**DOI:** 10.3389/frai.2025.1614608

**Published:** 2025-07-11

**Authors:** G. Sumathi, M. Uma Devi

**Affiliations:** Department of Computing Technologies, SRM Institute of Science and Technology, Kattankulathur, Chengalpattu, Tamil Nadu, India

**Keywords:** image inpainting, probabilistic framework, generative adversarial network, prior estimation, Papoulis–Gerchberg algorithm, MAP estimation

## Abstract

Addressing inpainting challenges in high-resolution images remains a complex task. The most recent image inpainting techniques rely on machine learning models; however, a major limitation of supervised methods is their dependence on end-to-end training. Even minor changes to the input often necessitate retraining, making the process inefficient. As a result, unsupervised learning approaches have gained prominence in image inpainting. State-of-the-art methods, particularly those using generative adversarial networks (GANs), have achieved promising results. However, generating photorealistic outputs for high-resolution images with arbitrary large-region masks remains difficult. Inpainted images often suffer from deformed structures and blurry textures, compromising quality. Additionally, building a model capable of handling a diverse range of images presents further challenges. These challenges are addressed by proposing a novel probabilistic model that utilizes picture priors to learn prominent features within StyleGAN3. The priors are constructed using cosine similarity, mean, and intensity, where intensity is computed using the improved Papoulis–Gerchberg algorithm. The image is reconstructed using the probabilistic maximum *a posteriori* estimate. Variational inference is then applied to obtain the optimal solution using a modified Bayes-by-Backprop approach. The model is evaluated on 70,000 images from the Flickr-Faces-HQ, DIV2K, and brain datasets and surpasses state-of-the-art techniques in reconstruction quality.

## 1 Introduction

Reconstructing images from incomplete or degraded data is a challenging and important problem in computer vision. This issue is solved by the process of inpainting, the goal of which is to fill in the degraded or damaged areas that may be caused due to degeneration or manipulation. Inpainting makes an effort to fill those damaged areas with pixels from the same or a related image, ensuring that the newly created pixels blend seamlessly with the current image and give the image a realistic appearance. Since the filling is not performed randomly, image inpainting is often viewed as an imputation task, where missing data are estimated based on observed content. Imputation is an approach for restoring the unavailable data with some alternative value to maintain the data in its complete form. The inpainting approaches are basically classified as diffusion, patch and learning-based methods and other methods, such as the Naive Stokes method, Fast-Marching method, etc. (Ghorbanzade et al., 2020; Nabizadeh et al., 2020). Diffusion or patch-based inpainting aims to fill the empty portions with the pixel values of the surrounding regions because images are truly a spatial collection of pixel values (Goodfellow et al., 2014; Nazeri et al., 2019; Zhang et al., 2018). In the Naive Stokes method, the edges of the images are recognized as continuous, and so the pixels near the edges are taken into account while reconstructing the missing portions. The Fast-Marching method, which used the weighted sum of the pixels from a nearby pixel, was another method that sought to solve the reconstruction problem.

Although the Naive Stokes and Fast-Marching methods produced conceptually sound results, the outputs were often affected by noise and lacked clarity (Goodfellow et al., 2014; Nazeri et al., 2019; Zhang et al., 2018). The easiest way to handle this problem is to map a single image to numerous images that are accessible as external data. A perfect match for the missing areas is sought by searching a sizable library of diverse photos. This approach, introduced by Hays and Efros (2007), enabled the retrieval of the most suitable patch from a large image database for accurate filling of the missing region. The reconstruction appeared realistic since it was done using semantic reconstruction of the image. Finding databases containing a variety of photographs is a difficulty with this endeavor, though. These techniques require significant computer resources to find the missing pixels without any prior knowledge of the image. This suggests that these techniques might help handle photos with fine lines. Therefore, due to the heavy computation and semantic filling, the usual approach may disastrously fail when it comes to inpainting larger regions. This necessitates a technique that will solely populate the image depending on the comprehension of the one that is provided. As a result, learning-based approaches can now be used in the inpainting problem.

Learning-based methods focus on replacing the missing pixels, often known as “mask pixels,” with the surrounding pixels. The most pertinent neighboring pixel can only be fitted to the masked pixels using this procedure. Since the invention of the generative adversarial network (GAN; Zhang et al., 2020), image reconstruction has been done using many methods with various goals. The results of the inpainting study for replacing the objects are clear from Zeng et al. (2020); Bau et al. (2020); Ntavelis et al. (2020b); Yu et al. (2018); Zhang et al. (2021). Similarly, employing supervised or unsupervised techniques, the goal of filling landscapes or altering the content of a particular picture has produced notable results. A big part of choosing the best pixel for the masked regions was played by encoders. Different variations of variational autoencoders, such as VQ-VAE (Higgins et al., 2017) and VAE (Van den Oord et al., 2016b), have shown good results when it comes to image generation tasks in the quest for a superior inpainting image. The use of various auto-regressive models, such as PixelCNN, PixelRNN, NeutralODE, Glow, and RealNVP, yielded positive results for inpainted high-resolution images (Van Den Oord et al., 2016a; Chen et al., 2018; Kingma and Dhariwal, 2018). The training distribution has a significant impact on the quality of the high-quality images produced by the autoregressive models. These results, which were produced using several types of GANs, are acceptable in terms of how effectively they handle tiny mask regions and produce high-quality images. The robustness and need for retraining of conventional and learning-based techniques, however, is the real problem. The difficulties mentioned above, in general, are more narrowly focused, and the reconstruction is a model-specific inversion. Given these challenges, a comprehensive technique that can handle any corruption process and a mask that is not constrained to a narrower or rectangular region are required. Along with this, there is also a continuous shifting of input that might occur very often in specific domains and may become troublesome because of the limited computational resources. Leveraging the pre-trained models to carry out the reconstruction tasks becomes a mandate. Despite the remarkable success of deep generative models, particularly GANs and diffusion models, a common drawback observed in high-resolution inpainting is the averaging effect (Ledig et al., 2017). This phenomenon arises when the generator attempts to reconstruct missing content by minimizing a global loss across multiple plausible completions, often resulting in blurry, semantically diluted outputs. Such averaging effects are particularly pronounced in the case of large, irregular, missing regions, or complex textures. Pre-trained models are inherently limited in their capacity to generate diverse inpainted results under such conditions due to their single-point inference mechanism.

To address this, the proposed study introduces a probabilistic framework for image inpainting that treats the task as a Bayesian inverse problem, thereby explicitly modeling uncertainty over the latent space. By leveraging meaningful priors constructed from cosine similarity, mean intensity, and directional features and optimizing over the posterior distribution using a modified Bayes-by-Backprop approach, the model can sample from a structured latent space and produce sharper, contextually accurate reconstructions without relying on mode averaging. This approach directly mitigates the averaging problem and leads to robust results across diverse datasets.

The theory of considering inpainting as an inverse problem and solving it using priors has been tested in several studies (Pan et al., 2021; Asim et al., 2020; Lugmayr et al., 2020; Jalal et al., 2021a,b). The vast majority of methods rely on Langevin dynamics, which emphasizes single-point losses and sluggish mixing, to find solutions. There are questions about using GANs to compile a distribution of patches for a corrupted image and using models in a probabilistic framework to do inpainting. This research suggests a probabilistic technique for inpainting using StyleGAN3. The following are some of the study's key contributions:

- To estimate meaningful prior images by using cosine similarity, mean and intensity as prior terms.
- To minimize the maximum *a posteriori* (MAP) estimate, image inpainting is carried out in a principled probabilistic framework utilizing StyleGAN3 and the corrupted image as input.
- In order to estimate the Gaussian distribution over the latent vector, the Bayes-by-Backdrop approach is used with a modification.
- Along with the typical state-of-the-art techniques, the method is validated using the Flickr Face dataset, the DIV2K dataset, and a brain medical dataset.

The remaining study is structured as follows: the goal of Section 2 is to provide a general overview of the inverse problem and how it is addressed in inpainting. A thorough literature review of the various deep learning and generative methods that aid in creating a better-reconstructed image is provided in the next section. Section 4 deals with problem formulation. The system model and architecture are both framed in Section 5. The experimental setup, result analysis using the inpainted results, and quantitative assessment are all covered in Sections 6 and 7, respectively. The final section brings the study to a close.

## 2 Preliminaries

### 2.1 Background: inverse problem

As the inverse problem pertains to something that cannot be measured directly, inpainting, when considered the recovery of missing pixel values in an image, is synonymously correct. Let there be an image with a total of *I*_*pixels*_ and *M*_*pixels*_. The *M*_*pixels*_ are the total number of missing pixels from the image. Unknown *M*_*pixels*_ require the discovery of data obtained by calculations known as *C*_*pixels*_. There is a relationship between *M*_*pixels*_ and *C*_*pixels*_ that can be formally stated as shown in Equation 1:


(1) $$\begin{array}{l} C_{pixels}=H\;\left(M_{pixels}\right) \end{array}$$

The “forward operator” is the operator that connects the known and unidentified quantities. Since the smaller variance in missing pixels does not affect its missing values, the forward operator is now well-posed. Since *C*_*pixels*_ is an image, the forward operator *H* is a measurement matrix that could be formed because of some operation like downsampling. This poses a dimension reduction, so an ill-posed problem is posed, which needs to be addressed using certain techniques. If inpainting is done carefully, it can be used to restore any missing or damaged portions of an image. An inpainting operation should provide a reconstruction that closely resembles the original pixels. Most often, mathematically, the reconstruction of the corrupted images is best described as a linear inverse problem, as shown in Equation 2:


(2) $$\begin{array}{l} y=\;F\left(x\right)+n \end{array}$$

where *x* is the ground truth image, *F* is the forward operator or measurement process (e.g., masking, blurring, transformation), *y* is the observed corrupted image or measurement, and *n* is the additive noise. Here, xεX represents the original (clean) image in the image domain, and yεY is the observed corrupted image obtained *via* the forward mapping F:X → Y, which models the corruption process (e.g., masking, blurring). The term *n* denotes measurement noise or degradation artifacts introduced during acquisition. This formulation reflects a general inverse problem setup, where the task is to recover *x* from the corrupted observation y.

As a result, it is simple to draw a comparison between the inverse problem and the problem of inpainting. Understanding the many methods used to tackle the inverse problem is crucial for formulating the proposed problem. The goal of this part is to give a general overview of the background information for the numerous inverse problem solutions that can be utilized to control inpainting.

#### 2.1.1 Inverse problem-solving using an analytical approach

Analytical operation, as its name suggests, seeks to define the forward operator given in Equation 1 as a well-known mathematical operator and thereafter employ the proper inversion operator. Let there be a 2D image represented as *f* (*x*,*y*) and the corrupted image as *g*(*x*′,*y*′), then the forward relation between these two can be mentioned as a convolution operator. The relation between the forward and inverse operators can be mathematically represented, as shown in Equation 3:


(3) $$\begin{array}{l} g\left(x^{\prime },y^{\prime }\right)=\;H\left[f\left(x,y\right)\right]+n\left(x^{\prime },y^{\prime }\right) \end{array}$$

where *g*(*x*′,*y*′) is the corrupted image, *f* (*x*,*y*) is the original image, *H* is the forward operator representing the inpainting process, and *n*(*x*′,*y*′) represents noise or artifacts introduced during the inpainting process.

Fourier methods are the well-known mathematical operators utilized in an analytical approach. The inverse Fourier transform, alone or in combination with other transform techniques, is frequently used to tackle the issues. The difficulty here is the applicability of the forward model in real-world scenarios if it is referred to as either of the Fourier transforms. This strategy appears to be practically impossible because it can only produce better outcomes if the data are accurate and full (Jackson et al., 1991).

#### 2.1.2 Inverse problem-solving using a generalized approach

In this case, it is presumable that the inverse problem can be solved by more than one forward model. This method is comparable to the analytical method, which transforms the full image first and then approximately approximates the resulting image. The key elements of the image are contained in the modified image matrix representation, which can be utilized to recreate the missing sections roughly. A variety of techniques can be employed to create this approximation matrix, but principal component analysis, singular value decomposition, and the least squares method are the most widely used techniques (Huan et al., 2010).

This method calls for the definition of a set containing every potential solution, from which one potential solution is chosen to use a predetermined set of criteria. The applied criteria are as straightforward as obtaining the bare minimum normalized solution. The employment of an iterative algorithm to optimize the established criteria is the primary distinction between the analytically based and generalized techniques. This is the general concept underpinning the deep learning architectures that contribute to a larger degree to the resolution of the inpainting problem.


(4) $$\begin{array}{l} g\left(x^{\prime },y^{\prime }\right)\;=\;\left\{\;f{\left(x,y\right)}^{+}:Hf^{+}\right\} \end{array}$$

As mentioned in Equation 4, if *g*(*x*′, *y*′) is the corrupted image which requires the best possible solution for the original image, *f*(*x, y*) is a potential solution for the original image, Hf+ denotes the forward operation applied to the potential solution *f*(*x, y*), and so the solutions for this type of inverse problem can be obtained, as shown in Equation 5:


(5) $$\begin{array}{l} f^{+}=H^{+}g \end{array}$$

where *f* ^+^ represents the reconstructed version of the original image *f* . It is the result of applying the inverse operation *H*^+^ to the corrupted image, *g*. *H*^+^ This is the inverse operator of the forward operator *H*. It is responsible for undoing the effects of the forward operation and attempting to recover the original information and *g* is the corrupted image.

#### 2.1.3 Inverse problem-solving using model-based approach

This approach again gets its roots from the classical analytical approach where a corrupted image *g*(*x*′, *y*′) is obtained with the assistance of the forward model *H* and the original image *f*(*x, y*). Since this is a model-based approach, the prediction of the unknown particles in the image *g*(*x*′, *y*′) can be deduced with a deterministic or probabilistic approach. The most common deterministic approach is regularization, which adds up a regularization parameter along with the general inverse problem framework.


(6) $$\begin{array}{l} f_{r}=\;\arg_{f}min\;\left[\frac{1}{2}\|g-I_{\Omega }f{\|}^{2}\right]+\;\lambda \text{Ψ}\left(f\right)\; \end{array}$$

where *f*_*r*_ is the reconstructed image (final solution), f is the candidate solution in the image space, g is the observed corrupted image, *I*_**Ω**_ is the binary mask operator that selects the known (non-missing) pixels, ||·|| denotes the ℓ2-norm, Ψ(*f*) is the regularization function or prior (e.g., smoothness, sparsity) and λ is the regularization parameter controlling the strength of the prior.

In this energy minimization framework, the goal is to estimate a plausible reconstruction *f*_*r*_ by minimizing a cost function Φ(*f, g*), which balances data fidelity and regularization. The first term $\|g-I_{\Omega }f{\|}^{2}$ enforces consistency between the reconstruction *f* and the known pixels in the observed image *g*. The operator *I*_Ω_ acts as a binary mask, selecting only the observed (non-missing) pixel locations. The second term λΨ(*f*) imposes a prior or regularization (e.g., smoothness or semantic coherence), with λ controlling the trade-off between fidelity and prior constraints. Finding a unique solution is challenging since the regularization term can only be limited to smoothness or sparsity. The extremely limited knowledge that is available to present an absolute answer to the missing pieces is another issue to be concerned about when attempting to employ a deterministic approach.

The drawbacks of the deterministic approach force us, then, to view the solution to the inverse problem from a new angle. Having an account of the prior estimations that enable the computation of uncertainty is the perspective that can be particularly helpful in addressing the problem of inpainting. The priors can be effectively used to obtain a meaningful fill rather than just some values to fill in the vacant pixels by guessing the hyperparameters. Exactly this is what is frequently done when probabilistic methods are used to construct a posterior, which internally combines priors and likelihood. The base idea in the probabilistic approach is derived from the Bayes theorem that is mentioned in Equation 7:


(7) $$\begin{array}{l} P\left(f|g,H\right)\;=\;P\left(g|f,H\right)P\left(f|H\right)\;/\;P\left(g|H\right) \end{array}$$

where *P(f|g,H)* is the conditional probability of the original image, *f* given the corrupted image, *g* and the forward operator, *H. P(g|f,H)* is the likelihood of the data, *P(g|H)* is the maximum likelihood and *P(f|H)* is the prior. The prior knowledge has provided us with a general understanding of how the inverse problem can be resolved. The probabilistic technique appears to be adequate and can yield superior results after analyzing all the approaches and realizing that the inpainting can be considered an inverse problem.

## 3 State-of-the-artworks: neural networks and inverse problem

**Figure 1:**
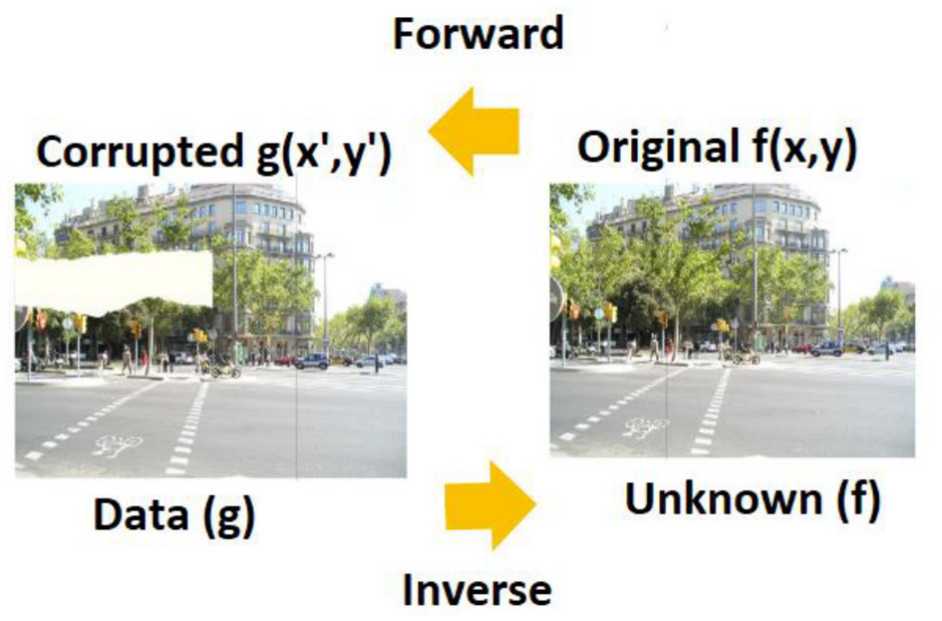
Forward and inverse operations as convolution and deconvolution. DIV2K Dataset by Timofte et al., licensed under CC BY 4.0, https://data.vision.ee.ethz.ch/cvl/DIV2K/.

From the background information, irrespective of the method chosen, it is evident that multiple solutions are at hand. The procedure that will help to extract the specific solution is to identify what can be fitted into the problem for visualizing and identifying if the decision was made correctly or not. As mentioned, the flow involves the combination of several steps that are depicted in Figure 1.

**Figure 2:**
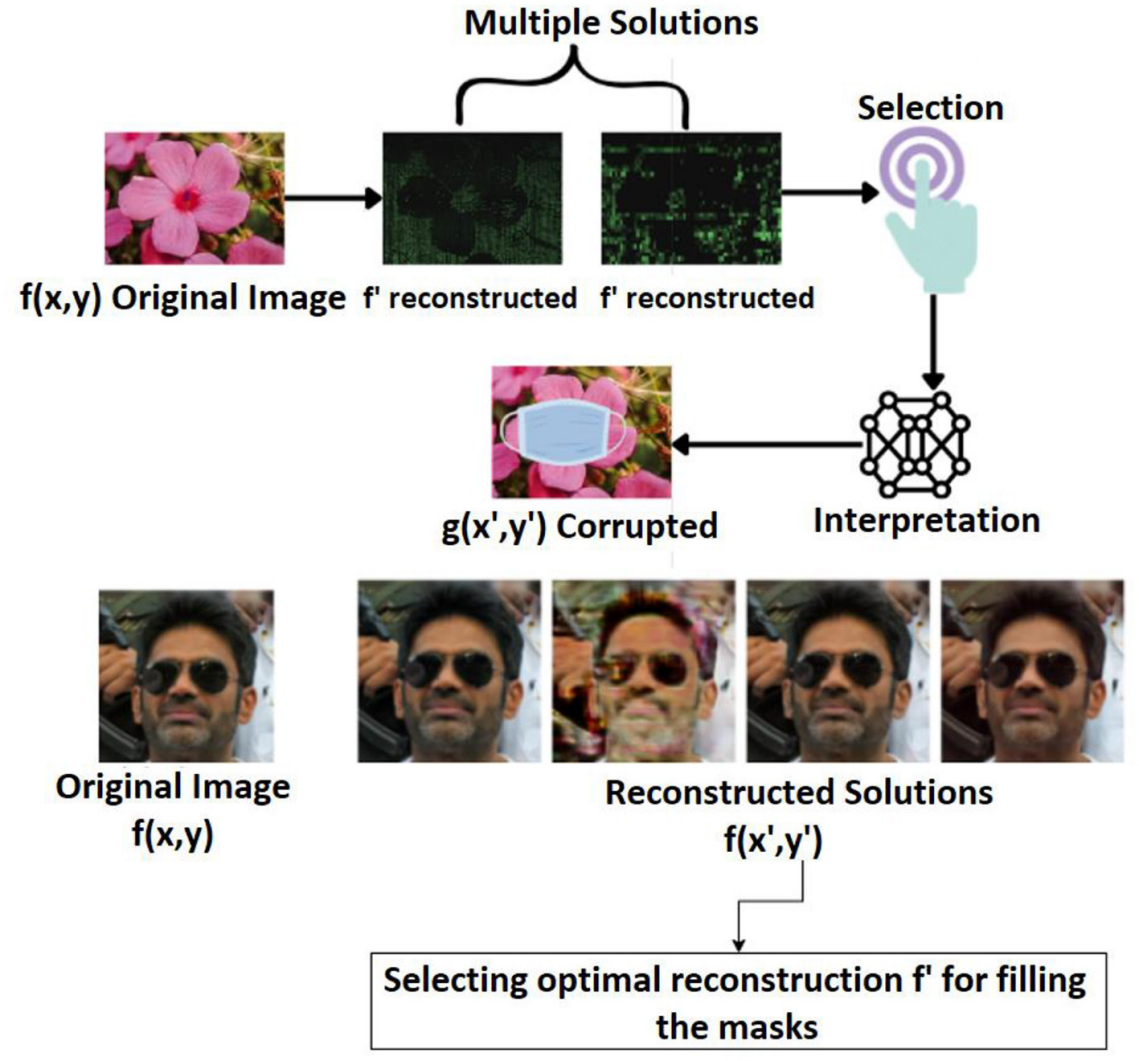
Incorporating automated models into inverse problems. Facial images reproduced from Flickr-Faces-HQ (FFHQ) Dataset by NVIDIA Corporation, licensed under CC BY-NC-SA 4.0, https://github.com/NVlabs/ffhq-dataset.

To fill in the missing pixel in the corrupted image from the original image, numerous solutions are generated, from which just the interesting solution needs to be selected, as shown in Figure 2. Here, *f'* represents the initial intermediate reconstruction obtained before prior-guided optimization. It serves as the starting point in the inverse problem pipeline. Interpreting the chosen solution and improving predictions calls for some prior knowledge. In order to bring things together, segmentation, interpretation, and decision-making are required. With the aid of artificial intelligence and machine learning, all these steps can be completed simultaneously. A literature review is thus offered in this section to pinpoint the precise neural network model that may be applied to resolve the inpainting problem.

### 3.1 Deep neural networks

When a single-layer neural network is used to try to solve inpainting as an inverse issue, it takes the easier route without even having a forward model. A collection of inputs S: (f,g)k = [1,2,3; K] and outputs S: (f,g)k = [1,2,3; K] make up the model. The neural network learns this full set of inputs using an iterative learning method, which is then applied to build a new solution with a new instance of image (Ciresan et al., 2011; Gilton et al., 2019; Gong et al., 2020). After defining a loss function, the neural network generally uses an optimization method to try and optimize the loss function. Although this approach can be extended to numerous layers of neural networks, its main drawback—the lack of a forward model—means that it is only effective for smaller datasets.

### 3.2 GANs and inpainting

The development of GANS (Goodfellow et al., 2014) significantly contributed to the improvements made in the area of picture inpainting. As indicated in Pathak et al. (2016), a context encoder is employed to obtain semantically accurate image inpainting. Initially, the encoder–decoder architecture was used as the generator. Semantic image regeneration takes the image's surroundings into account in order to come up with a good theory for the missing pieces. When it comes to semantics and conditioning the image with its surroundings, some similar experiments (Hui et al., 2020; Liu et al., 2020; Ntavelis et al., 2020a) have produced encouraging results. Diverse types of bespoke convolution approaches are incorporated within the reconstruction pipeline to inpaint a photo-realistic image, ensuring the generation appears authentic. In order to properly direct the inpainting according to the mask, various convolutions, including dilated (Iizuka et al., 2017), partial (Liu et al., 2018), and gated (Yu et al., 2019), are accessible in the pipeline. When these approaches were examined attentively, the outcomes appeared photorealistic and generated outstanding outcomes in the context of temporal synthesis. The results, however, are not particularly unique when it comes to meaningfully synthesizing the image.

Building not only photorealistic but also sane semantic reconstructions is another way to use GANS in the context of inpainting. Various studies focus on meaningful picture reconstruction when inpainting is viewed as an inverse problem. A general Bayesian framework was offered in this study (Adler and Öktem, 2018) for solving the painting inverse problem both with and without a loss function. The number of projections is a key factor in determining how successfully to recreate the mask conceptually. In some cases, especially when trying to recreate the medical images, defining a broader projection set is impossible. This is simply handled in Bora et al. (2018), which mentions a variation of GAN that labels measurements as genuine or synthetic rather than focusing on differentiating between real and created images.

To prevent the variance seen between the generated and observed images, a different GAN variant called MimicGAN (Anirudh et al., 2020) has been utilized. This kind of variation is feasible because some photographs have unneeded cropping, etc. This necessitates optimizing the latent vector, which is essentially the focus of this study. The latent projections are initially obtained by Mildenhall et al. (2021) and Abdal et al. (2020) using Image2StyleGAN++, and these projections are then used to tackle a variety of image editing issues, such as inpainting.

The diffusion model is another model that is frequently employed in the context of inpainting to find precise pixels for the missing regions. Diffusion models used in inpainting attempt to obstruct the training image by adding noise. The noise eventually obliterates every aspect of the image until it is entirely noise. Then, using the diffusion model to help with denoising, the reverse procedure is carried out until a clear image is obtained. In Sohl-Dickstein et al. (2015); Song et al. (2020), the issue of inpainting is addressed with the aid of diffusion models and differential equations. Meng et al. (2021) makes the case for the application of a denoising diffusion model. The frequency information directs the fundamental concept of synthesizing the input image using a diffusion model. The inpainting task cannot be completed using this frequency-based picture synthesis since the missing regions do not have any frequency-related data. Reverse diffusion is another type of guidance. RePaint (Lugmayr et al., 2022) introduced a novel iterative denoising diffusion process with resampling, enabling it to fill in large and arbitrary missing regions by stochastically resampling intermediate states during inference. This strategy improves sample diversity and fidelity while maintaining global coherence in the generated image.

Recent advances in latent diffusion models (LDMs; Rombach et al., 2022; Yang et al., 2023) have shown remarkable promise in generating high-fidelity, semantically rich inpainting results. Unlike pixel-space diffusion models, LDMs operate in a compressed latent space using a pre-trained autoencoder, making them both computationally efficient and semantically aware. These models benefit from being trained on large-scale image-text datasets, such as LAION-5B, and can generalize well across a wide range of inpainting tasks.

LDMs have indeed demonstrated impressive capabilities in image generation, classification, and detection. However, when applied to image inpainting, particularly with large and irregular masks, they are still subject to certain limitations. Despite being pre-trained on rich datasets, the semantic averaging effect is a prominent issue, as the model tends to generate blurry or contextually ambiguous reconstructions for large missing regions. This is primarily because LDMs often rely on surrounding pixel context and global priors learned during pretraining, which may not be sufficient to capture the fine-grained structural and semantic details necessary to accurately fill large holes. This issue is also noted in VIPaint (Agarwal et al., 2024), where the authors attempt to mitigate this limitation through variational inference over the latent diffusion process.

To overcome this issue, the proposed study explicitly addresses this challenge by formulating inpainting as a Bayesian inverse problem and incorporating image-specific priors (cosine similarity, mean, and intensity) derived from the corrupted input itself. Although perceptual loss from pre-trained generative models (e.g., StyleGAN3) is utilized to shape the priors, the method does not rely solely on pre-trained latent representations. Instead, the reconstruction is guided through a posterior distribution that captures uncertainty and facilitates the sampling of diverse, coherent outputs. This approach helps overcome the averaging effect and achieves better reconstructions, particularly for large or semantically complex masked regions.

Another notable method, LaMa (Resolution-robust Large Mask inpainting; Suvorov et al., 2022), focuses on handling large and irregular holes with high-resolution input images. LaMa incorporates Fast Fourier Convolution (FFC) layers and a perceptual discriminator that guides the model to produce structurally and visually consistent completions. While LaMa performs well on inpainting tasks with large missing regions, its reliance on heavy architectural components and adversarial training can limit flexibility across domains.

To inpaint high-resolution images, Aggregated cOntextual-Transformation (AOT) GAN is employed in Zeng et al. (2022) to improve the content interpretation from far-off contexts and intensify the texture and composition of massively degraded regions. A pyramid of content and texture GANs are engaged in Cao et al. (2020) to fill the missing regions in low resolution and then improvise the textures in high resolution, respectively. To reconstruct arbitrarily damaged regions (Wang et al., 2021) incorporates a dynamic selection network and discriminates the known pixel regions from the damaged regions all around the network, thereby applying the knowledge in the known region to the fullest.

Inpainting studies involving explicit priors are also available in the literature, as mentioned in Xu et al. (2020), where a pre-trained holistically nested edge detection (Xie and Tu, 2015) is used to derive an incomplete edge map from the damaged image, which is then fully reconstructed using an edge inpainting network. This fully constructed edge map acts as a prior to reconstructing the completed image. The use of frequency domain priors (Roy et al., 2021) is another prior-based inpainting model, where frequency-related deconvolution is used to grasp the surrounding context, thereby restoring the high-frequency elements using a discrete Fourier transform. To inpaint images with complex structures and highly damaged regions, a semantic prior-based integrated contextual transformation network is deployed (Li et al., 2023).

The strategy to perform better inpainting has its roots in GAN and diffusion methods after a thorough analysis of numerous methodologies. A probabilistic strategy, in contrast to the methods discussed above, is employed in the proposed study with the aid of GAN. The technique can produce freeform inpaintings with high-quality images. Although any model, regardless of GAN or diffusion, can be imbued by the probabilistic approach that is used for reconstruction, the projected study focuses exclusively on StyleGAN and checking the same by utilizing the diffusion model is retained as a future scope.

## 4 System model

### 4.1 Problem formulation

The motivation for the proposed framework stems from a fundamental limitation observed in conventional pre-trained inpainting models: their tendency to produce averaged outputs when encountering multiple plausible completions. This arises due to deterministic inference and loss minimization over ambiguous latent representations. To address this, the inpainting task is formulated as a probabilistic inverse problem, where the objective is not to retrieve a single best guess, but rather to approximate the posterior distribution over possible clean images given the corrupted input *g*:


(8) $$\begin{array}{l} p\left(z|g\right)\propto p\left(g|z\right)\cdot p\left(z\right) \end{array}$$

This equation represents the posterior distribution over the latent variable *z*, conditioned on the corrupted image *g*, and is derived from Bayes' theorem. Here, *p*(*z*|*g*) is the posterior distribution, expressing how likely each latent code *z* is, given the observed corrupted image *g*. *p*(*g*|*z*) is the likelihood, representing the probability of observing *g* if *z* is the true latent representation. *p*(*z*) is the prior over the latent space [which may later be adapted to *p*(*z*|*P*) using structured priors]. Since *p*(*g*) (the marginal likelihood) is constant with respect to *z*, the equation is expressed in proportional form. This formulation enables the model to reason probabilistically about multiple possible reconstructions rather than collapsing to an average output, which is common in deterministic GAN-based methods.

In a direct problem, the objective is to predict the output of a system given complete knowledge of the input and model. In contrast, inverse problems start from observed outputs and aim to infer the underlying causes or inputs that generated them. Both inpainting and reconstruction fall under this inverse problem category, as they require estimating missing or latent information based on partial observations. Specifically, image inpainting refers to the process of filling in missing or corrupted regions of an image using contextual information from the surrounding known pixels. This task involves reasoning about the most plausible content that could occupy the missing region based on image structure, texture, and semantics—making it a classic example of an inverse problem.

Image reconstruction, on the other hand, typically involves recovering an image from its transformed representations, such as projections in computed tomography or compressed measurements in MRI. The goal here is to infer the original image from indirect observations, which again involves solving an inverse problem. In both scenarios, the underlying challenge is the same: deducing unknown or lost information from available measurements. The probabilistic framework adopted in this study aligns naturally with this formulation. We assume the reconstructed image is generated by a pre-trained model *G*(*z*), where *z* is a latent code:


(9) $$\begin{array}{l} x=G\left(z\right) \end{array}$$

To obtain the best reconstruction, we optimize a variational approximation of the posterior *q*(*z*|*g*) by minimizing:


(10) $$\begin{array}{l} L_{post}=KL\left(q\left(z|g\right)\|p\left(z|P\right)\right)+\;L_{perc}\left(G\left(z\right),x^{*}\right) \end{array}$$

where, $L_{post}$ is the posterior-guided variational loss, *KL*(•) is the Kullback–Leibler divergence between the approximate posterior and a prior *p*(*z*|*P*) constructed from perceptual features *P*, $L_{perc}$ is the perceptual loss, x^*^ is the ground truth image available during training.

To realize this, a Bayesian formulation using StyleGAN3 is proposed as the generative backbone, enhanced with carefully designed priors that encode semantic similarity, intensity structure, and mean consistency. These priors guide the variational inference over the latent space to favor coherent and realistic reconstructions. This probabilistic framework not only supports uncertainty modeling and diverse sampling but also avoids the collapse of blurred outputs that result from deterministic averaging. The design choices, from prior construction to MAP inference and optimization *via* Bayes-by-Backprop, are all motivated by this central goal, which is to eliminate semantic averaging and improve fidelity in large-region inpainting tasks.

If one attempts to solve the problem of inpainting using a probabilistic framework, the system model put forth is the general configuration that is used. Though originality is demonstrated in the previous generation process, the dependence of GAN on the pre-trained models is the main issue that is to be focused on. The Bayesian version of GAN reconstruction focuses on particular prebuilt datasets, such as FFHQ, DIV2K, brain, etc. Utilizing the probabilistic framework is still in its early stages for a diverse set of images. The following list of issues is the focus of attention:

- Apply inpainting to a variety of image sets and add a generative model into the suggested probabilistic framework.
- With an arbitrary mask, a better reconstruction must be accomplished.

**Figure 3:**
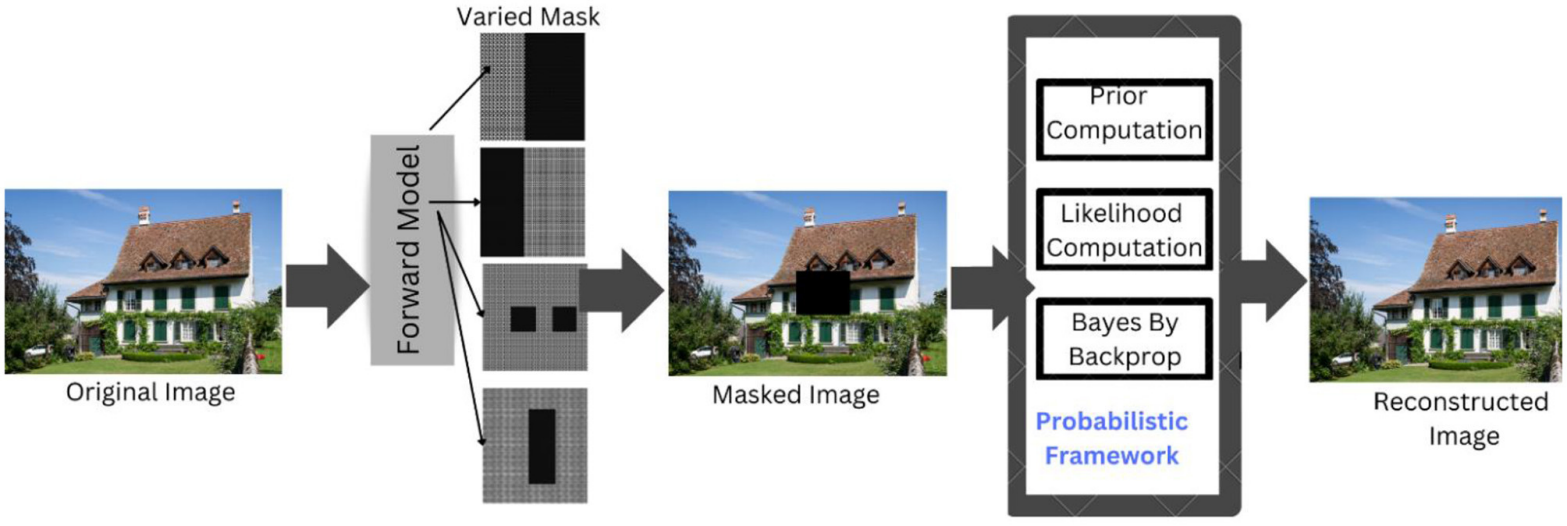
System model. DIV2K Dataset by Timofte et al., licensed under CC BY 4.0, https://data.vision.ee.ethz.ch/cvl/DIV2K/.

The proposed study suggests a probabilistic architecture made up of numerous components that aid in better picture reconstruction and inpainting. Figure 3 displays the broad perspective of the system model. The system employs a StyleGAN3, which serves as a forward model and implements a straightforward process to produce a corrupted image. Let the real image have the dimensions H and W, which represent the height and width of the image, respectively. The real image and the arbitrary mask are combined to create the corrupted image using the Hadamard approach. The damaged images produced by the processing of the forward model are then used as input for reconstruction to guarantee the accuracy of the inpainting results. The likelihood is formed by this Hadamard product. Then, meaningful picture priors are framed considering the cosine similarity, mean and intensity as prior terms. The intensity is obtained by customizing the Papoulis–Gerchberg algorithm. Furthermore, the reconstructed images are obtained through the MAP estimate, which is followed by the Bayes-by-Backprop for an optimal reconstruction solution.

### 4.2 Proposed probabilistic framework

**Figure 4:**
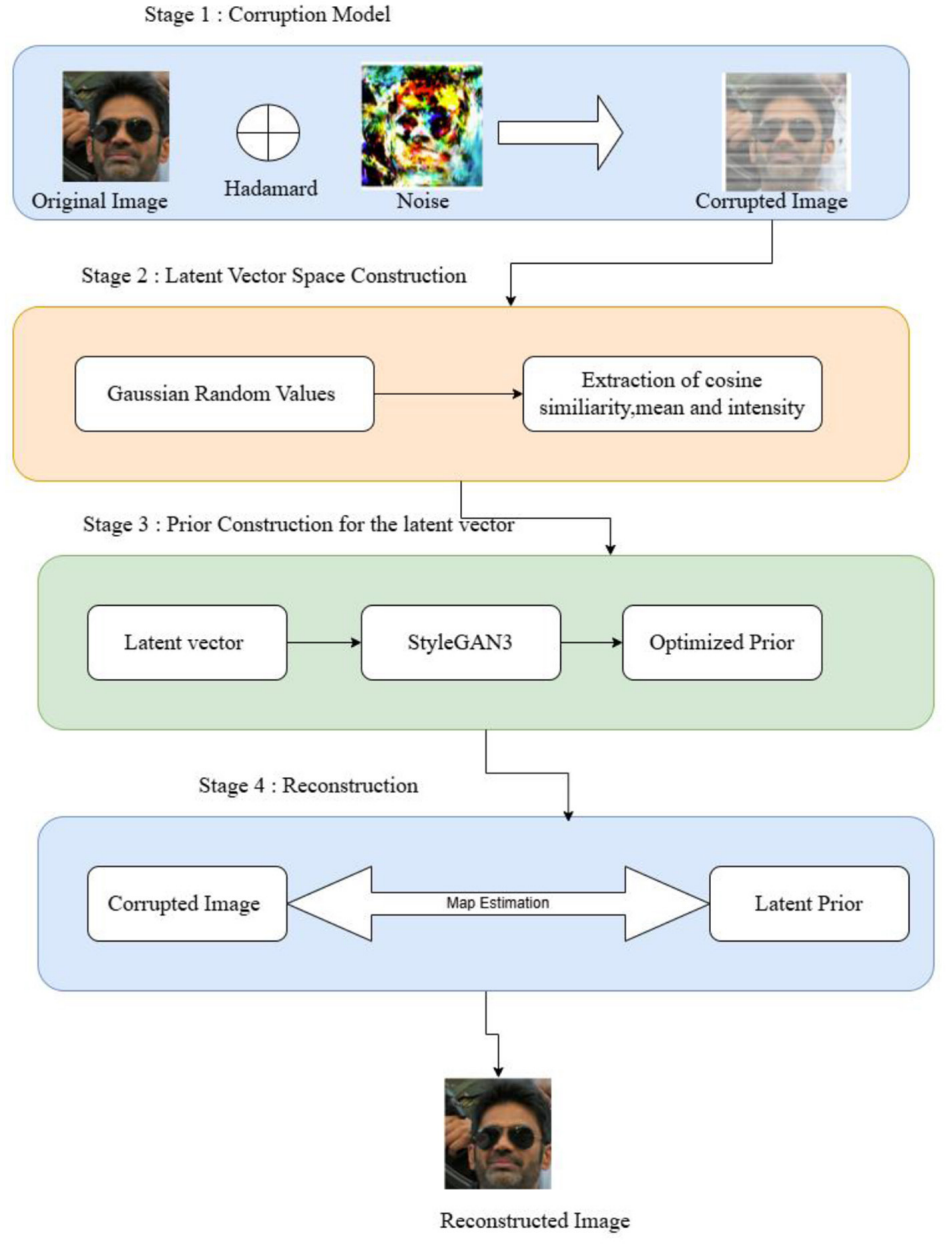
System workflow. Facial images reproduced from Flickr-Faces-HQ (FFHQ) Dataset by NVIDIA Corporation, licensed under CC BY-NC-SA 4.0, https://github.com/NVlabs/ffhq-dataset.

Having formulated the problem, this section briefs the proposed probabilistic framework general flow and the mathematical formulations of the method. Figure 4 shows the flow of the probabilistic framework.

Consider an image that is first exposed to noise, which causes the image to become corrupted. In this case, both the image and the noise parameters are known. It is described as *I*_*image*_, η_*pixel*_, η_*percept*_. *I*_*image*_ is the ground truth image, η_*pixel*_ and η_*percept*_ are the pixel noise and perceptual noise, respectively. Knowing the image and the conditions that result in a corrupted image are created as *I*_*corrupt*_ using the forward model. To effectively perform inpainting, this distorted image needs to be rebuilt with the correct pixels. To achieve this, the prior terms, such as the cosine similarity, mean and intensity written as α, β, γ, respectively, are required to construct the prior latent vector space, ω. After performing this process, the transformation is obtained as *I*_*Clean*_. The StyleGAN model's guidance is used to determine the variational parameters for approximating the Gaussian posterior, indicated as α_*v*_, β_*v*_, and γ_*v*_, which are then combined with the latent vector ω to carry out the reconstruction process. The inpainting results are referenced using the latent vectors *I*_*image*_ and *I*_*Clean*_. Algorithm 1 shows the overall working of the probabilistic framework.

**Algorithm 1:** Probabilistic framework.

**INPUT:** Corrupted Image *I*_*corrupt*_, Noise model parameters (η_*pixel*_, η_*percept*_), Prior parameters (α, β, γ)
**OUTPUT:** Clean Image *I*_*clean*_
**ALGORITHM:**
1. ***Prepare the corruption model:*** • Compute the corrupted image using the Hadamard product: • *I*_*corrupt*_ = *M* ⊙ *I*_*clean*_ 2. ***Initialize the Framework:*** • Initialize the latent space ω with random Gaussian values. • Extract initial priors (α, β, γ) based on cosine similarity, mean, and intensity. 3. ***Compute likelihood for each corrupted Image:*** • Use the forward model to simulate corruption • *P*(*I*_*corrupt*_|ω) = *Gaussian*(η_*pixel*_, η_*percept*_) 4. ***Prior computation:*** • Compute meaningful priors • *P*_ω_ = *P*_*cosine*_. *P*_*mean*_.*P*_*intensity*_ 5. ***Define loss function:*** • Compute total Loss • *Loss*_ω_ = *Loss*_*prior*_ + *Loss*_*Cosine*_ + *Loss*_*pixel*_ + *Loss*_*percept*_ 6. ***Optimize using Bayes-by-Backprop:*** • Iteratively update ω using probabilistic backpropagation • ω^*^=*Loss* 7. ***Reconstruct the image:*** • Generate the clean image, where G is the styleGAN3 model • $I_{clean}=G\left(\omega^{*}\;\right)$

#### 4.2.1 PRIOR modeling and estimation

As stated earlier, StyleGAN3 is not a prerequisite for using the probabilistic methodology suggested in the proposed work. This framework is being included since StyleGAN is only applicable to a subset of pre-trained models, which limits the type of data that can be used to evaluate the proposed model. The objective is to evaluate the framework using a variety of datasets so that it may be utilized with various generator models that can flow *via* gradients. Since the latent space of the StyleGAN, ω, which was previously mentioned, is known, it is necessary to mention this dimension to obtain *I*_*Clean*_. The StyleGAN3 is trained with ℝ^512^*18 in which the latent space comprises 18 latent vectors. These vectors form 18 resolution levels. Thus, the input specification of StyleGAN3 is ℝ^512^*18 which forms the ω when passed to a function that generates the output for the Flickr Face dataset is 1,024 × 1,024. Thus, the G(ω) forms the *I*_*Clean*_. Therefore, if the probability density function of the preceding term needs to be mentioned, it can be represented as indicated in Equation 11. The Jacobian determinant is used to describe the degree of transformation on the 18 levels with rotating, stretching, or morphing. The Jacobian determinant is the greatest way to explain the change in magnitude because it enforces 18 layers of a function within the resolution.


(11) $$\begin{array}{l} P(I_{Clean})=P(G(\text{ω}))=p(\omega )[\partial G(\omega_{1},\omega_{2},\omega_{3}....\omega_{18})]/\partial \omega ] \end{array}$$

The various resolution levels are obtained with the Jacobian determinant; now, the priors that are constructed need to be appropriate. This styleGAN3 latent vector space ω: = {ω_1_, ω_2_, ω_3_....ω_18_} needs to have meaningful priors. So, for this purpose, α, β, γ are defined by applying relaxation to the StyleGAN3. The prior terms defined are as follows:

- Cosine similarity prior, as mentioned in Menon et al. (2020), relaxes the default idea of StyleGAN3, which is that every vector is the same. This directional prior thus can bring significant differences between the ω_1_ and ω_2_, ω_2_, and ω_3_, etc. So, the two vectors are collinear.
- Mean prior to ensure that these latent vectors lie in the same region.
- Papoulis–Gerchberg, which is related to the intensity values, is another prior.

With the mention of the priors, now the probability density function is defined in Equation 12:


(12) $$\begin{array}{l} P_{\omega }=P_{cosine}.P_{mean}.P_{intensity} \end{array}$$

Here, the cosine similarity measures the angle between two vectors ω_*i*_ *and ω*_*j*_ and is defined as:


(13) $$\begin{array}{l} P_{cosine}=\frac{cos^{-1}\left(\omega_{i\;}\omega_{j\;}\right)}{\left\|\omega_{i}\|\|\omega_{j}\right\|} \end{array}$$

Additionally, μ, σ, and Σ represent the mean, standard deviation, and covariance of the latent variables, respectively. A normal distribution of the data is defined over the directional coordinates [0,2π], represented as K. Algorithm 2 outlines the computation of these priors.

**Algorithm 2:** Computation of priors.

**Input:** *I*_*corrupt*_ → Corrupted Image M → Mask ω, μ, σ, Σ → Latent variables α, β, γ → Prior Parameters **Output:** *P*_ω_ → Meaningful Prior **Algorithm:** 1. ***Find the target area:*** • Identify the masked region (M) and set the corresponding pixels in *I*_*corrupt*_ to zero. 2. ***Setting Meaningful Prior:*** • *Compute* *P*_*cosine*_: $$\begin{array}{l} P_{cosine}=\frac{cos^{-1}\left(\omega_{i\;}\omega_{j\;}\right)}{\left\|\omega_{i}\|\|\omega_{j}\right\|} \end{array}$$ • *Compute* *P*_*mean*_: Ensure consistency using a Multivariate Normal Distribution: $$\begin{array}{l} \forall_{i}\;\omega_{i}\;\sim N\left(\mu ,\Sigma \right) \end{array}$$ • *Compute* *P*_*intensity*_ *using the modified Papoulis*–*Gerchberg Algorithm:* ° Iterate over the entire image ▪ Make masked region zero ▪ Increase the known pixel value in the spatial domain 3. ***Output the computed prior:*** • *P*_ω_ = *P*_*cosine*_. *P*_*mean*_.*P*_*intensity*_

#### 4.2.2 Likelihood estimation

The inpainting problem is defined as an inverse problem and a probabilistic framework for the same is created that includes the prior and probability in the process of finding a solution to this inverse problem. It is necessary to produce the likelihood, as it is defined in Equation 6. The forward model for the corrupting process allows to create the likelihood model for inpainting. In this instance, all that is required is to multiply the clean image and mask pixels by pixels. Thus, the likelihood can be defined as in Equation 14:


(14) $$\begin{array}{l} P\left(I|I_{clean\;}\right)=\;P\left(I|G\left(\omega \right)\right) \end{array}$$

where G(ω), as specified in prior, is a Jacobian. Two types are used when considering the noise. Gaussian noise both at the pixel level and for perceptual perception. After the incorporation of the noise and likelihood, the likelihood model can be defined as in Equation 15:


(15) $$\begin{array}{l} P\left(I|op\;o\;G\left(\omega \right)\;\right)\;=P\left(I|f\;o\;\eta_{pixel\;}\;nf\;x\;nf\right),P\left(I|\Phi \;\eta_{percept\;}\;n_{f}x\;n_{f}\right) \end{array}$$

#### 4.2.3 Image inpainting estimation

It is now time to do the inpainting in order to produce a clear image after defining inpainting as an inverse problem using the prior and likelihood. Four loss terms are used in this definition of the objective function.

- *Loss*_*prior*_ is defined to be the prior loss on the latent vector, ω obtained by ${\left(\omega -\frac{\mu }{\sigma }\;\right)}^{2}$.
- *Loss*_*Cosine*_ is defined to be the collinearity loss on the latent vector, ω obtained by $\frac{cos^{-1}\left(\omega_{i\;}\omega_{j\;}\right)}{\left\|\omega_{i}\|\|\omega_{j}\;\right\|}$.
- *Loss*_*pixel*_ is defined to be the pixel-wise loss on the latent vector, ω obtained by *P*(*I*|*f o η*_*pixel*_ *nf x ñf*).
- *Loss*_*percept*_ is defined to be the perceptual loss on the latent vector, ω obtained by *P*(*I*|Φ* η*_*percept*_ *n*_*f*_*xñ*_*f*_).

Therefore, the Bayesian MAP estimate can be used to recover a clean image from a damaged one. Now that the likelihood and previous have been instantiated, the vector ω must be optimized. Alternating optimization is utilized for meaningful inpainting, and the main principle is to make the most of the data in ω.

With a distorted input image, the Bayesian estimate thus produces a clean image. Consequently, the goal function can be described in Equations 16, 17:


(16) $$\begin{array}{l} Loss_{\omega }\;=\;Loss_{prior\;}+\;Loss_{Cosine\;}+Loss_{pixel\;}+Loss_{percept} \end{array}$$

(17) $$\begin{array}{l} I_{clean\;}=\;argmax\;P\left(I|I_{clean}\right)P\left(I_{Clean}\right) \end{array}$$

Probabilistic by Backprop is now applied as many samples are collected for reconstruction. The posterior distribution over the weights P(θ|D) is what you wish to estimate in Probabilistic by Backprop. The network weights have a unique posterior distribution, and it is distinct from any specific data point. The probabilistic Backprop approach chooses to estimate this cost term by sampling, which has the benefit of supporting previous distributions with more complex priors. The modification done with the probabilistic Backprop method is used to estimate every data point. Gaussian estimation is done for every data point on the latent vector, which, in turn, can directly optimize the mean, covariance and standard deviation. Sampling is performed with noise, mean, covariance and standard deviation. Thus, the variational samples are computed by P(θ|D) in which θ are learning parameters to the true posterior to the latent vector ω. So, the variational samples are obtained using the Equation 18:


(18) $$\begin{array}{l} variation=P\left(D\right)=\left(\omega |\theta \right)\|\left(P\left(\omega |I\right)\right) \end{array}$$

where $P\left(\omega |I\right)=\frac{P\left(\omega |\theta \right)\log P\left(\omega |\theta \;\right)}{P(\omega )(P(I|\omega )}$

Through this, the variational parameters α_*v*_, β_*v*_, and γ_*v*_ are computed, in which the γ_*v*_ is a gamma distribution with the priors included.

## 5 Experimental setup

Having defined the framework for image inpainting this section briefly discusses the implementation details in terms of the dataset, model training, and optimization.

### 5.1 Dataset

The FFHQ dataset, the brain dataset, and the DIV2k dataset are used to assess the framework that has been proposed. Flickr-Faces-HQ (FFHQ) is a widely used benchmark dataset for GANs, consisting of high-quality images of human faces. The collection has 70,000 high-quality PNG photos, a size of 1,024 × 1,024 and a wide range of age, ethnicity, and image background. Additionally, it does a fantastic job of covering accessories like hats, sunglasses, and eyeglasses. The DIV2K dataset (Timofte et al., 2017), a high-quality dataset for image enhancement tasks with a 2K resolution, is employed to investigate the framework for a variety of image types. There are 800 photos in total in the training set. The brain dataset is taken into account to assess the framework in the most challenging medical imaging dataset. The five brain datasets included in this collection are ABIDE (Heinsfeld et al., 2018), PPMI (Marek et al., 2018), OASIS (Marcus et al., 2010), AIBL (Ellis et al., 2009), and ADNI (Jack Jr et al., 2008). A 90:10 split is employed for the FFHQ and brain dataset to train the StyleGAN3, with 90% of the data used for training and the remaining 10% for testing.

The test dataset comprises samples from three domains:

- Flickr-Faces-HQ (FFHQ): a 90:10 split was used, with 7,000 images (10% of 70,000) for testing
- DIV2K natural images: 100 standard test images were used.
- Brain MRI images: derived from five public datasets, ABIDE, PPMI, OASIS, AIBL, and ADNI, with a 90:10 split used for training and testing the StyleGAN3-based model.

Each mask type was applied to ~25% of the total test samples per dataset to ensure a balanced representation of occlusion scenarios. This balanced mask distribution allows us to assess the performance of the proposed method of varying levels of occlusion complexity and semantic structure. The average image area covered by each mask is as follows:

- Mask 1: covers ~15%
- Mask 2: covers ~25%
- Mask 3: covers ~50%
- Mask 4: covers ~50% of the image area

Although pre-trained models for StyleGAN3 supporting FFHQ, DIV2K, and brain datasets are available, they are not considered, and learning is performed on the Titan GPU using the StyleGAN3Config.

### 5.2 StyleGAN3 training

**Figure 5:**
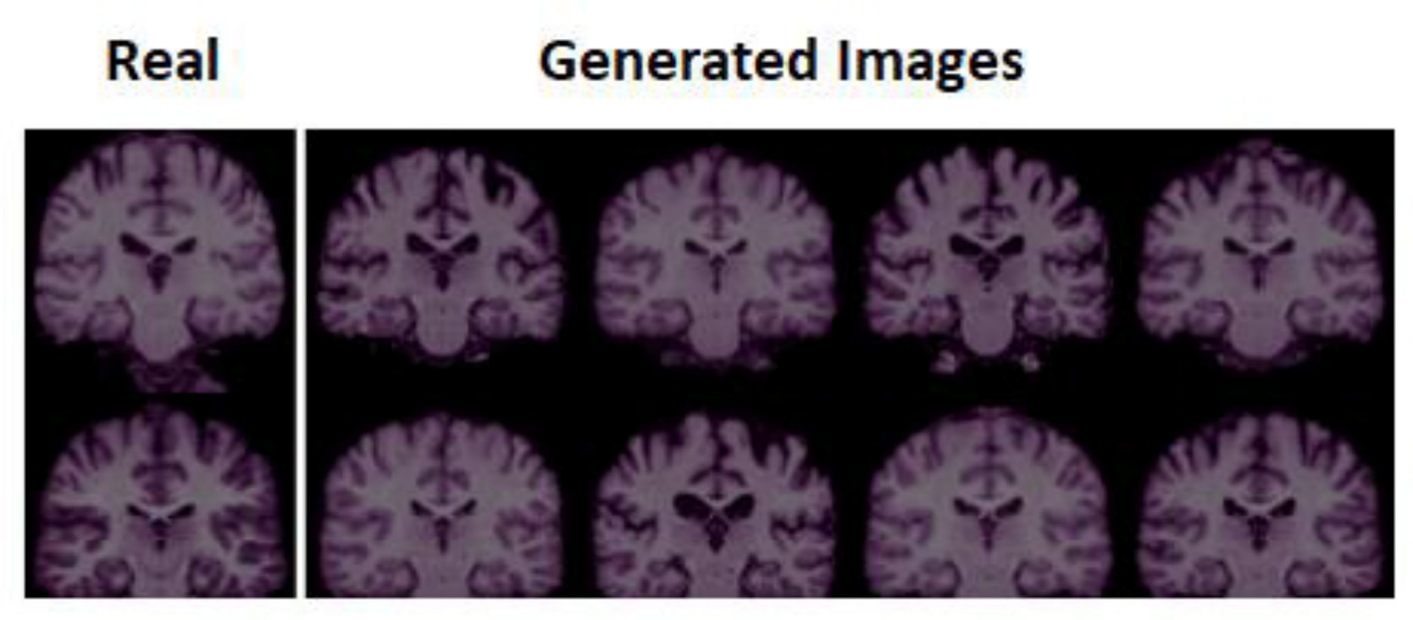
Reconstructed Images of brain dataset.

The training accuracy of the configured GAN is verified for any artifacts on all three datasets. The StyleGAN3 generator developed was trained on the brain dataset to produce uncrated images. Figure 5 shows the actual image on the left-hand side, taken at random from datasets along with the generated images of StyleGAN3 on the right-hand side. This training accuracy was very good considering all three datasets. It did not show any noticeable artifacts in the StyleGAN3-generated images. The picture quality is decent.

The proposed method employs between 32 and 34 s for 500 iterations of MAP inference on a 1,024 × 1,024 image, while it takes ~2.5 min for 500 iterations of fitting variational posterior parameters. After fitting the variational posterior, the model can instantly produce any arbitrary number of samples.

### 5.3 Model optimization

To optimize the loss function specified in Equation 12, the Adam Optimizer is used with a learning rate of 0.001. The answer to the inverse problem is acquired by gradually tweaking and optimizing the loss until a satisfactory solution is obtained, starting with the inversion produced by StyleGAN3. The inpainting output of the original StyleGAN was inadequate. omparison to the most recent techniques, the process of inpainting could be accomplished quite well by including the cosine, mean and intensity metrics into the latent space and removing noise from ω^+^. The smooth reconstruction of the images was made possible by the addition of cosine and perceptual loss, which made the inpainting appear realistic.

## 6 Results and discussions

Compared to state-of-the-art approaches, the inpainting inverse issue can achieve very impressive results when tested in a probabilistic environment. The outcomes from all three datasets are analyzed using the inpainting and analytical results. Let us go over the findings in more depth.

### 6.1 Reconstruction analysis

The framework can deliver improved inpainting outcomes in most circumstances when the model is optimized. The proposed method is evaluated using different masks across multiple datasets. To ensure correct inpainting, the input image is cropped appropriately. The outcomes according to the dataset are discussed below.

#### 6.1.1 Inpainting using FFHQ dataset

##### 6.1.1.1 Mask 1

**Figure 6:**
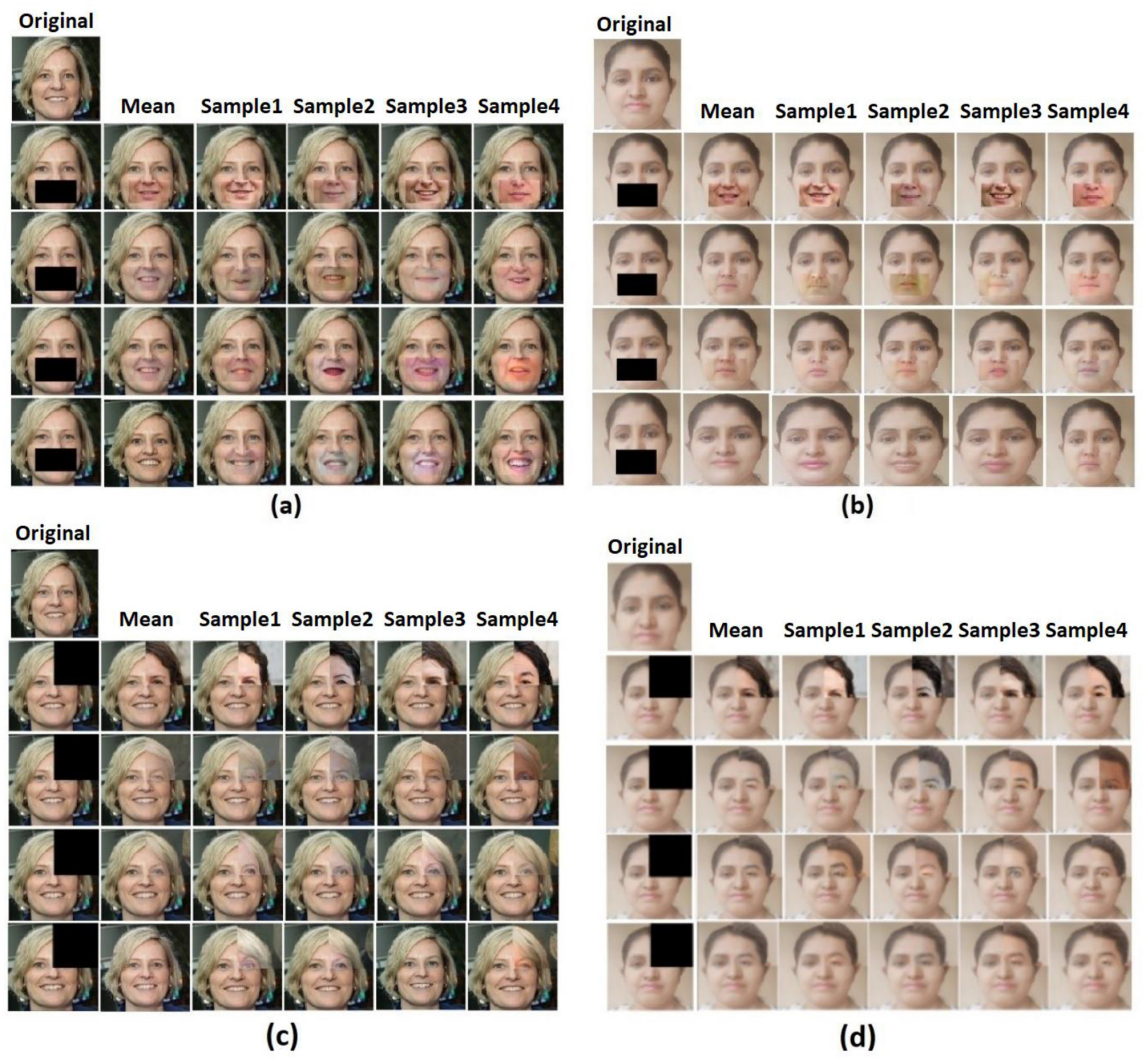
Sample iteration results: **(a)** image in FFHQ dataset with Mask 1, **(b)** real-time image with Mask 1, **(c)** image in FFHQ dataset with Mask 2, and **(d)** real-time image with Mask 2. Facial images reproduced from Flickr-Faces-HQ (FFHQ) Dataset by NVIDIA Corporation, licensed under CC BY-NC-SA 4.0, https://github.com/NVlabs/ffhq-dataset.

Mask 1 is designed to understand how the face reconstruction of the chin occurs so that the inpainting appears realistic. This result is compared to the results of the SN-Patch GAN for a state-of-the-art comparison. Figures 6a, b show a few iteration samples of the reconstruction results with the comparison of the original image. Here, each row represents a sample iteration. An image from the Flickr face dataset is used to analyze the results, and their outcomes are evaluated to see how closely the reconstruction resembled the images on which it was trained. The image labeled as original is the actual image, followed by the input-masked image and corresponding reconstruction sample results. It can be viewed that there are not many variations between the original image and some of the reconstructed results.

##### 6.1.1.2 Mask 2

The next mask, Mask 2, is square-shaped. Figures 6c, d show the inpainting results for the square mask for the image from the FFHQ dataset and the real-time image. The outcomes of the original and inpainted processes can be compared.

##### 6.1.1.3 Mask 3

**Figure 7:**
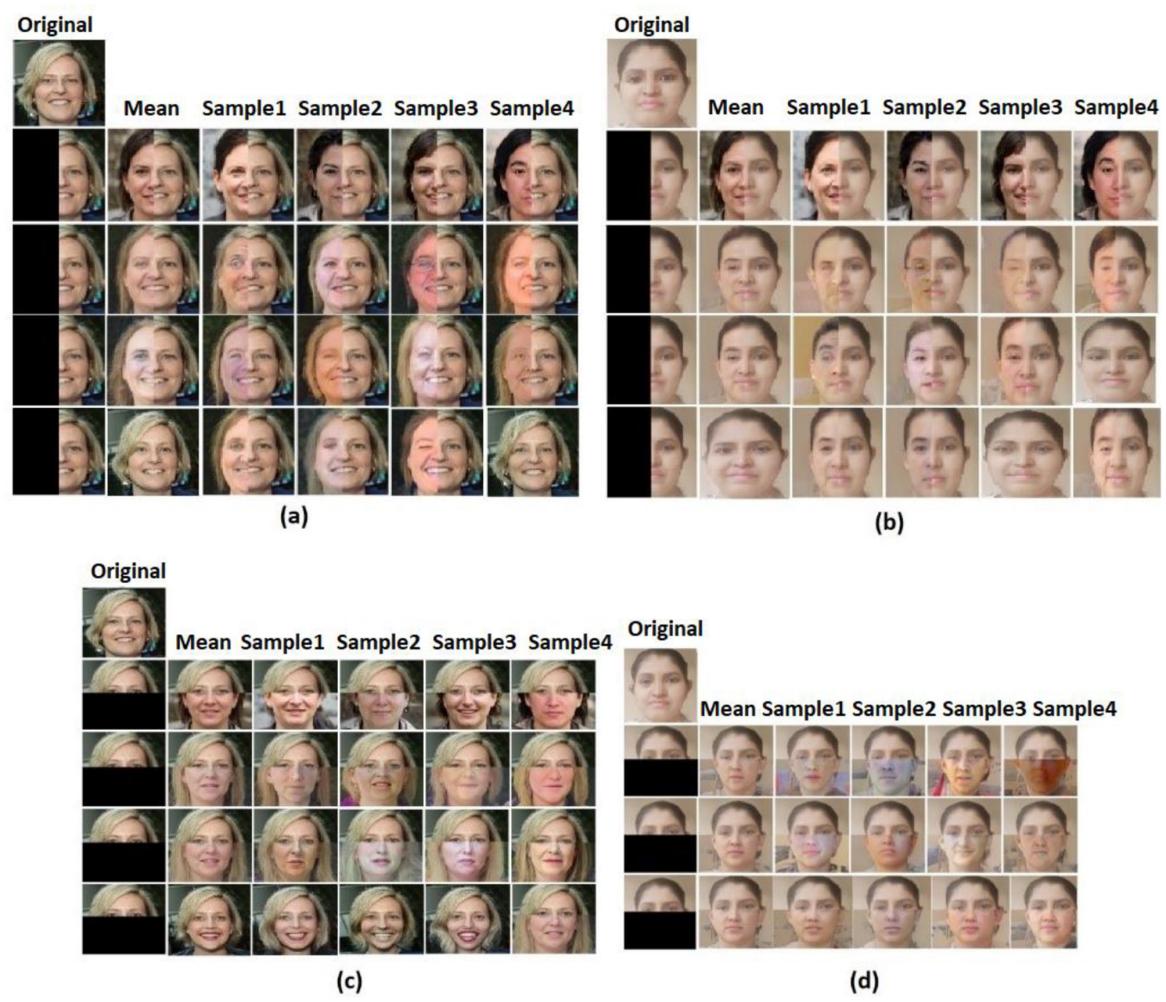
Sample iteration results: **(a)** image in FFHQ dataset with Mask 3, **(b)** real-time image with Mask 3, **(c)** image in FFHQ dataset with Mask 4, and **(d)** real-time image with Mask 4. Facial images reproduced from Flickr-Faces-HQ (FFHQ) Dataset by NVIDIA Corporation, licensed under CC BY-NC-SA 4.0, https://github.com/NVlabs/ffhq-dataset.

The inpainting results when there is an extremely large mask is one of the significant achievements in this study, so the next mask, Mask 3, that has been tried is a large vertical rectangular mask that covers almost half of the image. As shown in Figures 7a, b, this framework has outperformed even in the extremely larger-sized mask.

##### 6.1.1.4 Mask 4

Like Mask 3, the next mask, Mask 4, is extremely large, but it covers the bottom half of the image. Figures 7c, d show the sample iterations of Mask 4 applied on the FFHQ image and the real-time image.

**Figure 8:**
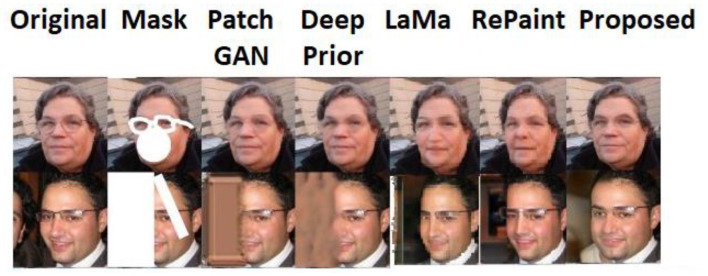
Qualitative comparison of the proposed and the state-of-the-art works, PatchGAN (Yu et al., 2019), deep prior (Ulyanov et al., 2018), LaMa (Rombach et al., 2022), and RePaint (Lugmayr et al., 2022) with arbitrary masks. Facial images reproduced from Flickr-Faces-HQ (FFHQ) Dataset by NVIDIA Corporation, licensed under CC BY-NC-SA 4.0, https://github.com/NVlabs/ffhq-dataset.

With different kinds of masks applied to the FFHQ dataset, the model performed exceptionally well. No matter how big or small the area, it had no impact on the inpainting. Even though we continued to attempt an arbitrary mask, the findings from the model were more advanced. The comparison results for arbitrary masks with the state-of-the-art models are displayed in Figure 8.

Compared to the SNPatch GAN, which has demonstrated excellent performance in the inpainting challenge, the results produced for inpainting by the probabilistic framework are quite good. When trying to inpaint an image that contains more than one face (say two faces), as shown in the second row of Figure 8, the state-of-the-art models fail, whereas the probabilistic framework comparatively produces a quite acceptable result.

#### 6.1.2 Inpainting using brain dataset

**Figure 9:**
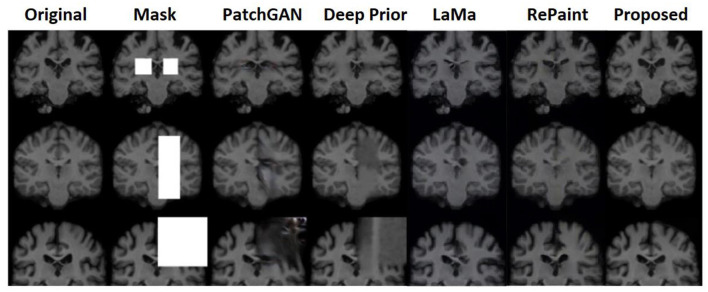
Comparative analysis of a probabilistic framework with PatchGAN (Yu et al., 2019), deep prior (Ulyanov et al., 2018), LaMa (Rombach et al., 2022), and RePaint (Lugmayr et al., 2022) on brain dataset.

On the brain dataset, the model with several mask pattern patterns was also successfully recreated. For all the different types of masks, the framework performed well, and the state-of-the-art comparison also showed good results for this dataset as well. Figure 9 shows the comparative results on the brain dataset with the PatchGAN and deep prior models.

**Figure 10:**
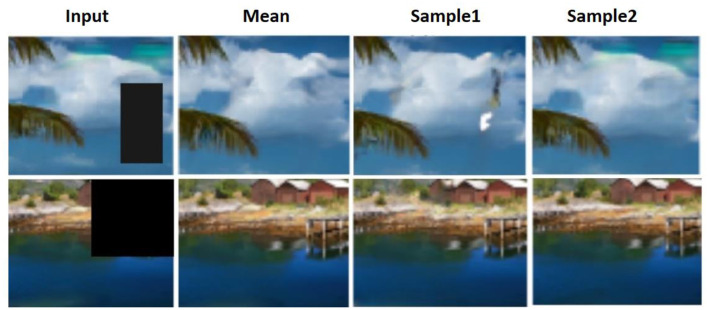
Inpainting results for images from the DIV2K dataset. DIV2K Dataset by Timofte et al., licensed under CC BY 4.0, https://data.vision.ee.ethz.ch/cvl/DIV2K/.

The next part of the comparison is carried out with the DIV2K dataset to showcase the proposed framework performance with various types of images. The validation of DIV2k is conducted on various types of masks, and its reconstruction results are seen. The inpainting outcomes for the diverse collection of photos from the DIV2k dataset are shown in Figure 10.

So, three separate datasets with every conceivable mask were subjected to a full inpainting study. As can be seen from Figures 6–10, the inpainting performed admirably both on its own and when compared to the state of the art.

### 6.2 Quantitative evaluation

To compute the difference between the original image and the inpainted image, RMSE and PSNR metrics are chosen for evaluation. A smaller RMSE and a higher PSNR reflect a closer similarity between the original and the inpainted image. The RMSE and PSNR values of the proposed probabilistic framework (PF) are confirmed with the SNPatchGAN (SNP GAN; Yu et al., 2019), deep prior (Xie and Tu, 2015), RePaint (Lugmayr et al., 2022), and LaMa (Rombach et al., 2022) after 50 unseen photos of varying resolutions used for the quantitative evaluation. When the resolutions are higher than 64, the overall observation of RMSE and PSNR shows a lower value. When given unseen photos, the performance of the model degrades just a little; however, the styleGAN3 generator is unable to produce high-resolution images for unseen images. Results are good even at this early level because the model created is not particular to any mask or corrupting process. Table 1 shows the comparative results of the RMSE and PSNR values for all three datasets when four different masks are applied and evaluated. The probabilistic framework consistently outperforms and remains competitive with diffusion-based and other models, particularly under large and irregular mask scenarios.

**Table 1:** Evaluation of probabilistic framework using RMSE and PSNR metrics with other popular models for inpainting.

**Dataset**	**RMSE**					**PSNR**				
	**PF**	**SNP GAN**	**Deep prior**	**RePaint**	**LaMa**	**PF**	**SNP GAN**	**Deep prior**	**RePaint**	**LaMa**
FFHQ	24.28	30.75	33.62	26.12	25.91	20.32	19.05	17.36	19.74	19.86
DIV2k	14.82	22.56	18.43	16.90	17.04	25.64	21.01	19.27	23.22	22.97
Brains	9.25	26.74	25.32	10.80	11.52	29.97	20.47	20.38	27.60	26.42

To evaluate the structural similarity and perceptual similarity between the original and inpainted images, SSIM and LPIPS metrics are chosen. Table 2 shows the comparative results of the SSIM and LPIPS values for all three datasets. The higher the SSIM and the lesser the LPIPS represent better inpainting.

**Table 2:** Evaluation of probabilistic framework using SSIM and LPIPS metrics with other popular models for inpainting.

**Dataset**	**SSIM**					**LPIPS**				
	**PF**	**SNP GAN**	**Deep prior**	**RePaint**	**LaMa**	**PF**	**SNP GAN**	**Deep prior**	**RePaint**	**LaMa**
FFHQ	0.86	0.8	0.8	0.82	0.81	0.17	0.26	0.28	0.21	0.23
DIV2k	0.95	0.83	0.81	0.89	0.88	0.11	0.22	0.24	0.18	0.20
Brains	0.86	0.78	0.75	0.82	0.80	0.07	0.2	0.22	0.12	0.14

**Figure 11:**
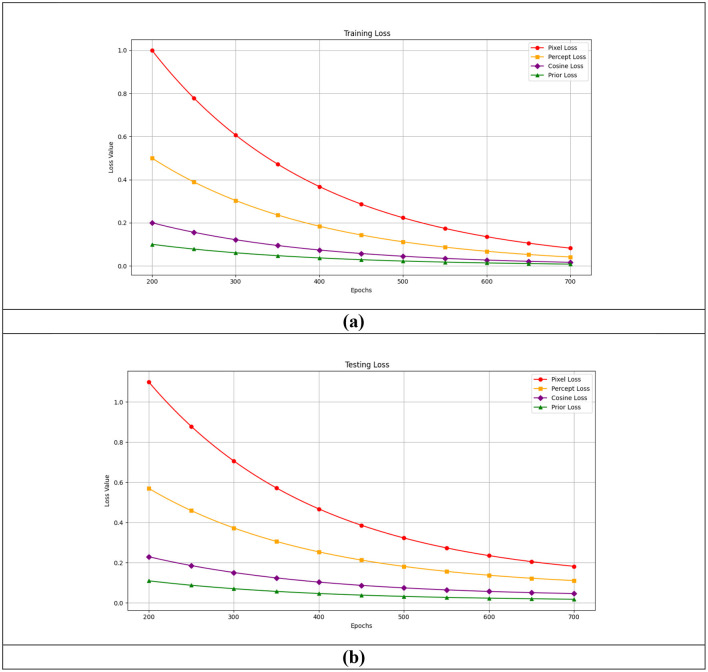
The **(a)** training and **(b)** testing loss plot for the brain dataset.

Following a thorough quantitative analysis, it was discovered that the Peak Signal-to-Noise Ratio (PSNR), Structural Similarity Index Measure (SSIM), Root Mean Square Error (RMSE), and Learned Perceptual Image Patch Similarity (LPIPS) values for all three datasets were highly excellent and outperformed the other comparison models. These values are better with the probabilistic technique, regardless of the type of mask. The training and validation plots for the brain data in Figure 11 consider each loss that made up the overall loss. The training and validation phases clearly differed when observed with values, and the curve was stable after it reached the point of stability, demonstrating the success of the two phases. There were no overfitting or underfitting difficulties, and the results for each dataset looked to be the same.

Though the training and validation plots for other datasets, such as FFHQ and DIV2K, are available, their training trends were observed to be similar, making additional plots redundant. This similarity is achieved because, despite the differences in image characteristics across datasets, the model effectively learns meaningful representations through prior and likelihood-based inpainting, ensuring stable convergence. The use of well-defined loss functions and hyperparameters across datasets further contributes to the uniform training behavior. This consistency indicates that the model generalizes well across diverse image domains, demonstrating its robustness in handling various inpainting tasks. This selection ensures clarity and conciseness without compromising the analysis. However, the results for all datasets have been thoroughly evaluated and discussed in the quantitative analysis.

### 6.3 Advantages of the proposed probabilistic framework

The probabilistic framework offers several distinct advantages:

- **Probabilistic modeling:** by adopting a Bayesian framework, the proposed method models uncertainty and captures multiple plausible reconstructions.
- **Latent priors:** the use of cosine similarity, mean, and intensity priors ensures the reconstructed features are semantically and structurally coherent.
- **Model generalizability:** unlike most inpainting methods that retrain for each domain, the proposed framework generalizes across FFHQ, DIV2K, and brain datasets without model-specific tuning.
- **Flexible integration:** although StyleGAN3 is used for evaluation, the framework is agnostic to the generator and can be adapted to other architectures, including future diffusion-based GAN hybrids.
- **Inference speed and applicability:** while the proposed framework avoids retraining across domains and mask types, it relies on iterative optimization during inference *via* Bayes-by-Backprop. This results in longer inference times (e.g., ~8–10 s per 512 × 512 image) than single-pass GAN or diffusion models. However, this trade-off is offset by the flexibility to handle diverse and unseen image types without any model-specific adjustments. Future studies may explore amortized inference or hybrid approaches to reduce latency for interactive applications.

These characteristics enable the proposed method to outperform both traditional and state-of-the-art models in terms of both perceptual quality and structural integrity.

### 6.4 Ablation study

Two criteria are used to conduct the ablation analysis. The use of hyperparameters is appropriate because this technique is a crucial design component. The sensitivity to hyperparameters for the probabilistic model was determined during ablation. The losses specified in Equation 13 are modified along with the prior, pixel, perception, and cosine parameters to assess their sensitivity to the model's performance. The corresponding LPIPS and MSE values are computed after one of the values is changed, while the other parameters are held constant in order to determine this. This ablation study is performed using 10 images from the brain dataset with a resolution factor of 64. These hyperparameters are set to constant values, and a single parameter is varied and obtained. The results are tabulated in Table 3. It is inferred that the parameters are not sensitive enough to affect the performance of the model after thoroughly analyzing the LPIPS and RMSE.

**Table 3:** Ablation study of hyperparameters.

**Pixel Loss**			**Percept Loss**			**Cosine Loss**			**Prior Loss**		
**Set values**	**LPIPS**	**RMSE**	**Set values**	**LPIPS**	**RMSE**	**Set values**	**LPIPS**	**RMSE**	**Set values**	**LPIPS**	**RMSE**
10^−7^	0.69	49.75	10^7^	0.6	38.96	10^−^4	0.55	31.52	10^−^4	0.53	33.69
10^−6^	0.5	35.23	10^6^	0.55	34.56	10^−^3	0.5	33.08	10^−^3	0.52	35.82
10^−5^	0.6	38.93	10^5^	0.58	36.67	10^−^2	0.59	37.67	10^−^2	0.55	39.17
10^−4^	0.57	36.33	10^4^	0.52	34.19	10^−^1	0.63	43.92	10^−^1	0.73	63.92
10^−3^	0.58	36.78	10^3^	0.59	36.52	10^0^	0.68	73.21	10^0^	0.78	82.21

### 6.5 Discussion on mode collapse and output diversity

A common limitation in generative inpainting, particularly with GAN-based methods, is mode collapse, where the model tends to generate repetitive or homogeneous textures across different reconstructions. This issue becomes particularly significant when repairing large or semantically complex masked regions, leading to a loss in output diversity and realism.

The proposed probabilistic framework mitigates this issue by treating the inpainting task as a Bayesian inverse problem, enabling the estimation of a posterior distribution over the latent space. Rather than producing a single fixed reconstruction, the model performs variational sampling through a modified Bayes-by-Backprop approach, allowing for multiple plausible and semantically valid outputs. The incorporation of structured priors—based on cosine similarity, intensity, and mean—ensures that each sampled solution is guided by both local structure and global context.

This mechanism effectively reduces the risk of convergence to limited patterns or repeated textures. The diversity across sample reconstructions is clearly illustrated in Figures 6a, b, where multiple iteration outputs show perceptually different but semantically consistent completions. Furthermore, in Figure 8, the model demonstrates superior handling of multi-object scenes (e.g., two-face example), where competing models collapse to incomplete or incoherent reconstructions, while the proposed approach maintains output variability and semantic integrity.

To support the claim of improved output diversity, we computed the intra-sample LPIPS score, which measures perceptual differences between multiple reconstructions of the same input. The analysis was conducted on 10 masked test images, covering all four mask types. Each input was inpainted using five different latent samples, and the average LPIPS diversity score was 0.312 ± 0.03. This demonstrates that the proposed framework produces perceptually diverse outputs and effectively mitigates mode collapse.

## 7 Conclusion and future study

Several strategies for resolving the problem of inpainting were researched and determined when it was successfully defined as an inverse problem. It was determined that the probabilistic framework is the best option for resolving the issue since it offers us the freedom to select the previous models and the choice to consider all the hyperparameters. With different masks and different data, multiple inpainting tasks were carried out using the suggested probabilistic framework. On three different datasets, the task of inpainting was illustrated using arbitrary masks. A unique advantage is that the proposed probabilistic framework is independent of StyleGAN3, thereby enabling the proposed framework to be executed with other generative and deep learning models. One of the main challenges in inpainting is reconstructing images with large and arbitrarily shaped damaged regions. Adopting the prior instances generated by StyleGAN3 to estimate the probability of obtaining the clean image assists in inpainting large masked regions. The proposed framework achieves promising results compared to state-of-the-art methods. The ablation study reveals the importance of the hyperparameters in the probabilistic methodology, thereby insisting on the sensitivity of the hyperparameters when tuning.

Extending the study to more intricate corruption models will be the focus of future efforts. Furthermore, the proposed probabilistic framework can be extended by incorporating LDMs as the generative backbone. LDMs operate in a compressed latent space and leverage pre-trained knowledge from large-scale datasets, offering powerful priors for high-fidelity generation. By integrating LDMs within the current Bayesian formulation, it would be possible to jointly model uncertainty and semantic consistency in a more scalable and expressive manner. This fusion could enable diverse and perceptually rich inpainting, particularly for complex and high-resolution image domains, while still preserving the benefits of posterior-guided reconstruction.

## Data Availability

The original contributions presented in the study are included in the article/supplementary material, further inquiries can be directed to the corresponding author.
